# Addition of 20-kDa PEG to Insulin Lispro Alters Absorption and Decreases Clearance in Animals

**DOI:** 10.1007/s11095-016-2014-1

**Published:** 2016-08-15

**Authors:** Mary Pat Knadler, Tri-Hung Nguyen, Kristina Campanale, Michael J. De Veer, John M. Beals, Shun Li, Ryan Hansen, Angela Siesky, M. Dodson Michael, Christopher J. H. Porter

**Affiliations:** 1 Lilly Research Laboratories, Eli Lilly and Company, Indianapolis, Indiana 46285 USA; 2 Drug Delivery, Disposition and Dynamics, Monash Institute of Pharmaceutical Sciences, Monash University, 381 Royal Parade, Parkville, Victoria, 3052 Australia; 3 Department of Physiology, Faculty of Medicine, Nursing and Health Sciences, Monash University, Victoria, 3800 Australia; 4 ARC Centre of Excellence in Convergent Bio-Nano Science and Technology, Monash Institute of Pharmaceutical Sciences, Monash University, Parkville, Victoria, Australia

**Keywords:** insulin peglispro, lymphatic absorption, sheep model

## Abstract

**Purpose:**

Determine the pharmacokinetics of insulin peglispro (BIL) in 5/6-nephrectomized rats and study the absorption in lymph duct cannulated (LDC) sheep.

**Methods:**

BIL is insulin lispro modified with 20-kDa linear PEG at lysine B28 increasing the hydrodynamic size to 4-fold larger than insulin lispro. Pharmacokinetics of BIL and insulin lispro after IV administration were compared in 5/6-nephrectomized and sham rats. BIL was administered IV or SC into the interdigital space of the hind leg, and peripheral lymph and/or serum samples were collected from both LDC and non-LDC sheep to determine pharmacokinetics and absorption route of BIL.

**Results:**

The clearance of BIL was similar in 5/6-nephrectomized and sham rats, while the clearance of insulin lispro was 3.3-fold slower in 5/6-nephrectomized rats than in the sham rats. In non-LDC sheep, the terminal half-life after SC was about twice as long vs IV suggesting flip-flop pharmacokinetics. In LDC sheep, bioavailability decreased to <2%; most of the dose was absorbed via the lymphatic system, with 88% ± 19% of the dose collected in the lymph after SC administration.

**Conclusion:**

This work demonstrates that increasing the hydrodynamic size of insulin lispro through PEGylation can impact both absorption and clearance to prolong drug action.

## INTRODUCTION

Adequate and stable basal insulin levels are a critical component of diabetes therapy because they regulate hepatic glucose output, which is essential for proper maintenance of glucose homeostasis during the diurnal cycle and inter-meal periods and they help to minimize nocturnal hypoglycemia (1). Over the past ~75 years, various strategies have been employed to provide basal insulin therapy for patients with diabetes. Initially, insulin suspension formulations (e.g., Ultralente, Lente, and NPH) were used to delay the release of insulin from the subcutaneous (SC) site of injection (2–4). However, these early basal formulations poorly mimicked the flat physiologic profile of basal insulin, and in the case of NPH, had a short duration of action (<24 h) (5). The introduction of insulin analogs and derivatives (e.g., insulin glargine, insulin detemir) coupled with an appropriate formulation strategy has shifted insulin therapy from suspension-based products to solution-based products. Moreover with insulin detemir,(6) this solution-based product reduced the variability observed in the suspension-based products (7–9). However, these approaches fall short of being true once-daily basal insulin substitutes in all patients (10,11); thus, there remains a need for a new generation of basal insulin products with sufficient duration of action to suffice as true once-daily basal insulin therapy.

Current basal insulin treatments attempt to prolong the residence time of insulin in the body and thus, prolong the duration of action. Decreasing clearance is a potential way to prolong the residence time of insulin in the body. Because the kidney plays a role in the elimination of insulin, one strategy for prolonging the duration of action of insulin could be decreasing renal clearance by decreasing the glomerular filtration and/or catabolism of insulin that occurs in the kidneys. A 5/6-nephectomized rat model can be used to study the impact of renal clearance on the overall clearance of a molecule. In this model, the kidney function of the rat is decreased by approximately 83%, which would be sufficient to affect the systemic clearance of a molecule that is prone to renal clearance, such as insulin.

In addition to slowing clearance, another potential mechanism to increase the duration of action is to prolong absorption from the SC injection. After SC administration, a protein therapeutic at the injection site can be absorbed via the blood capillary system and/or the peripheral lymph system (12). When a therapeutic protein dose is absorbed via the blood capillary system, it is immediately present in the systemic circulation compared with absorption into peripheral lymph where the protein is transported through the central lymphatic system and then drains into the systemic blood circulation via the right and left subclavian veins (13,14). The route of protein absorption after SC administration has been correlated to molecular weight of the protein (14–16) and a function of the endothelial basement membrane structure. Microvascular beds are primarily composed of continuous endothelial cell-cell interfaces characterized by tight adherens junctions that are impermeant to molecules the size of albumin (MW = 69 kDa), but, allow permeability for lower molecular weight molecules (17,18). Lymphatic capillaries have an incomplete basement membrane made from a single layer of overlapping endothelial cells and no tight junctions, leaving the vessel permeable to larger molecular weight macromolecules (14,19). Alternatively, in the case of insulin degludec, protein absorption has been delayed by the formation of multi-hexamers in the subcutaneous depot from di-hexamers in the pharmaceutical formulation upon diffusion of phenol from the formulation following injection (6).

Although SC drug administration is widely used, the bioavailability of drugs administered via this route remains highly variable, with few studies measuring the extent of uptake into the lymphatic system (20). Other factors that may affect the rate of absorption of a SC injection beyond the molecular weight of the molecule are the charge on the molecule surface and the formulation; as these factors affect the movement of the molecule in the extracellular matrix.

A lymph duct-cannulated (LDC) sheep model has been used in several studies to measure lymphatic absorption. This model provides an understanding of lymphatic absorption and bioavailability of drugs as a function of molecular weight (21–24). The LDC sheep model allows for simultaneous collection of peripheral lymph and sampling of blood over an extended time. The samples from the LDC sheep are compared with intact, non-LDC sheep to elucidate the relationship between lymphatic transport and drug bioavailability (20).

The LDC sheep model has been used to identify the route of absorption of SC-administered, commercially formulated human insulin. Charman and colleagues tested the hypothesis that hexameric-formulated human insulin is absorbed by the capillary system and to a lesser degree by the lymphatic system.(13) In LDC sheep, 17.3% of the insulin dose was recovered in the peripheral lymph demonstrating that lymph absorption was a contributor to the overall insulin bioavailability (13). Over multiple studies, Porter and colleagues have demonstrated that molecular weight (MW) correlates with the proportion of the dose absorbed (14–16).

The objective of these studies was to determine what impact hydrodynamic size of basal insulin peglispro may have on the absorption and clearance of insulin. This report characterizes the impact of the addition of a 20-kDa PEG to insulin lispro, the route of absorption in a lymph duct-cannulated sheep model, and renal clearance in a 5/6-nephectomized rat model.

## MATERIALS AND METHODS

### Materials

The PEGylation, purification, quantification, and formulation and stability of BIL used in animal studies are well defined in the US patent for BIL (25). The PEG reagent was purchased from the NOF Corporation (Tokyo, Japan). Eli Lilly and Company provided the BIL used in the studies. In summary, preparation of BIL was achieved using basic pH conditions at 4°C. Concentrated activated-PEG (para-nitro-phenylcarbonate activated methoxy-PEG) solutions were prepared in water at 4°C. Reactions were initiated by titration of the PEG solutions into the insulin lispro using a syringe pump (New Era Pump Systems, Inc., Model NE-300, Serial 232508 [Farmingdale, NY]) at a rate of addition varying from 0.5 to 3.5 mL/min. The reaction solution pH was maintained at ~11.0 by addition of base (NaOH or tribasic sodium phosphate). The reaction temperature was maintained at 4°C. At the completion of the reaction (0.5–4 h), the temperature was increased to 25°C and held for an additional 3.5 h. Reaction progress was monitored using a C8-based reversed-phase high performance liquid chromatography (RP-HPLC) method. The PEGylation reaction was stopped by adjusting the pH to 4.4 to 5. BIL was purified using either preparative C8-based RP-HPLC for small-scale preparations or cation exchange chromatography for large-scale preparations. The mono-PEGylated insulin lispro was eluted using either an acetonitrile-based gradient (RP-HPLC) or a NaCl-based step-gradient (cation exchange).

For the sheep studies normal saline and water for injection were sourced from Baxter Healthcare (Old Toongabbie, Queensland, Australia) and sodium heparin (1000 IU/mL) from Hospira (Mulgrave, Victoria, Australia). Sodium ethylenediaminetetraacetic acid (EDTA), TRIS base, sodium chloride, zinc oxide and *m*-cresol were purchased from Sigma-Aldrich (MA, USA). All other reagents used were of AR quality.

### Hydrodynamic Size Determinations

The hydrodynamic size of BIL, leptin, and insulin was determined using dynamic light scattering (DLS) measurements. Solutions were prepared from either freeze-dried powders or concentrated protein stock solutions (or both) of insulin lispro or PEGylated insulin lispro. Weighed-samples of freeze-dried powder were dissolved in 1x phosphate-buffered saline (PBS) to a final concentration of ~85 μM. Concentrated protein stock solutions were diluted ~20-fold in 1 × PBS buffer to achieve a final concentration of ~85 μM. Protein concentrations (OD_276_ (1 mg/ml) = 1 for a 1 cm cuvette) were measured by NanoDrop (Thermo Fisher Scientific, Waltham, MA, USA). All samples were filtered through Whatman Anatop10^™^ low protein-binding filters (Whatman, Kent, UK) before the DLS experiments. Solution viscosity was measured by an m-VROC® (RheoSense, Inc., San Ramon, CA, USA) viscometer and light scattering data were corrected using the measured viscosity.

DLS measurements were carried out at 25 ± 1°C at a 90° scattering angle using an ALV/CGS-3 Compact Goniometer system with ALV/LSE-5003 Light Scattering Electronics and Multiple Tau Digital Correlator (ALV®-GmbH, Langen, Germany). The laser operating wavelength was 633 nm. The apparent hydrodynamic radius R_h_ was estimated via the Stoke-Einstein equation (Eq. 1):

1 $$ {R}_h=\frac{k_BT}{6\uppi \upeta \mathrm{D}} $$

where, R_h_ is the apparent hydrodynamic radius (m)_,_ k_B_ is the Boltzmann constant (kg^●^m^2^/s^2●^K), T is the absolute temperature (K), η is the viscosity (kg/m•s), and D is the diffusion constant (m^2^/s). D_H_ is the apparent hydrodynamic diameter in nm or 2 ${R}_h\; in\; nm$. The viscosity measurement was performed with a solution concentration of 0.49 ± 0.02 mg/mL at 25°C. Viscosity mean and SD was determined from n ≥8 and reported as cP, (g/cm•s) = 10^−3^ × Pa•s (kg/m•s)

### Animals

Rodents were maintained in accordance with the Institutional Animals Care and Use Committee of Eli Lilly and Company and the National Institute of Health Guide for the Use and Care of Laboratory Animals. For the sheep models, all procedures were approved by the Monash University animal ethics committee and conducted in accordance to the Australian Code of Practice for the Care and Use of Animals for Scientific Purposes.

### 5/6-nephrectomized Rat Model

The 5/6-nephrectomized and sham male Sprague Dawley rats were obtained from Taconic Farms (Albany, NY, USA). Animals were housed two per cage in polycarbonate cages with filter tops. Animals were maintained on a 12/12 h light/dark cycle (lights on at 6:00 AM). All animals received water and LabDiet 5001 (PMI Nutrition International, Brentwood, MO, USA) *ad libitum*. Animals were monitored daily during the 5-week time frame that allowed progression of the disease state. Seven weeks post-surgery, blood was collected by tail clip into serum tubes for measurement of serum creatinine and blood urea nitrogen. Three days later, urine was collected for 24 h from animals housed in metabolism cages. Animals were block-randomized into groups based on body weight, 24-h urinary protein, and serum creatinine. On the day of study, animals were separated into individual cages, weighed, and bled by tail into a serum tube for the pooled group exposure sample at time, T = 0. Animals were then anesthetized with isoflurane and administered insulin lispro or BIL at 10 nmol/kg by penile vein injection at a dose volume of 1 mL/kg. Blood was collected via retro-orbital and/or tail bleeding at multiple time points after injection. At the last time point after injection, animals underwent CO_2_ inhalation, cardiac puncture to collect blood, and cervical dislocation. Insulin lispro exposure was determined using a radioimmunoassay (RIA) (EMD Millipore, St. Charles, MO, USA) and BIL exposure was determined using an enzyme-linked immunosorbent assay (ELISA) (Charles River, Senneville, Quebec, Canada).

### LDC and Non-LDC Sheep Model

Using a parallel group design, BIL was administered by single dose SC injection to non-LDC control sheep and to LDC sheep with a surgically placed popliteal lymph duct cannula (Table I). Blood was collected in each group, and lymph was continuously collected in the LDC sheep. BIL was also administered by bolus IV injection to a separate group of non-LDC animals for determining bioavailability and PK linearity.

**Table I:** Study Design and Treatment Groups for the LDC Study

Treatment Groups				
Group	Route	Dose nmol BIL/kg	Total Dose nmol/sheep	Matrix collected
LDC	SC	3	120	Serum/Lymph
LDC	IV	3	120	Serum/Lymph
non-LDC	SC	3	120	Serum
non-LDC	IV	3	120	Serum
non-LDC	IV	1	40	Serum
non-LDC	IV	0.5	20	Serum

Surgery was performed under aseptic conditions. Castrated male merino sheep (H.M. Barty & Sons Merino Stud, Robinvale, Victoria, Australia) weighing 31–48 kg were used. All animals had surgical cannulation of one jugular vein for blood collection via the insertion of a polyethelene cannulae (1.70 × 1.20 mm) (Microtube Extrusions, North Rocks, Australia), while animals in the LDC group had an additional 3 French heparin-coated polyurethane cannula inserted into the efferent popliteal lymph duct. Animals were prepared for surgery as previously described (26), and the efferent popliteal lymph duct was accessed and cannulated as previously described (13,20). Water and food was available *ad libitum* during recovery and sampling periods.

The sheep were dosed with a flat dose of insulin peglispro assuming a 40 kg sheep and then the exact weight based dose was calculated for each sheep. A single IV bolus dose of 20 nmol (0.5 nmol/kg), 40 nmol, (1.0 nmol/kg), or 120 nmol (3.0 nmol/kg) insulin peglispro (diluted as required with formulation diluent comprising 16.5 mM TRIS; 29 mM m-cresol; 128 mM NaCl; 0.39 mM zinc oxide (pH 7.3) in sterile water for injection) was administered into the jugular vein opposite the cannulated jugular vein used for blood collection. A single SC dose of 120 nmol (3.0 nmol/kg) insulin peglispro was injected using a 25 g needle into the interdigital space below the point of lymph cannulation for LDC sheep and the interdigital space of either leg for non-LDC sheep. Samples were taken from 3 to 5 sheep per dose group.

Blood samples (3 mL) were collected into 4 mL serum collection tubes (Becton, Dickinson and Company, Scoresby, Victoria, Australia) at the following time points (hour): predose, 0.0163 (1 min, IV only), 0.083 (5 min, IV only), 0.25 (15 min, IV only), 0.5, 1, 2, 3 (SC LDC only), 4, 6, 8, 10, 12 (all IV and SC LDC), 18 (SC non-LDC only), 30 (all SC), 39 (SC non-LDC only), 48 (all SC), 54 (all SC), 72 (all SC), 96 (SC LDC only), and 120 (SC LDC only) hours postdose. The jugular cannula was flushed with heparinized saline (50 IU/mL) between samples to maintain patency. Blood samples were allowed to stand at room temperature for up to 1 h and were then centrifuged for 10 min 6000 rpm to separate serum. Serum was stored at 4°C for up to 12 h and at −80°C for long term storage.

In LDC sheep, lymph samples were collected in pre-weighed tubes containing 10 μL/mL of 10% EDTA at the following time points: Predose, 0–1, 1–2, 2–3, 3–4, 4–5, 5–6, 6–7, 7–8, 8–9, 9–10, 10–11, 11–12, 12–24, 24–36, 36–48, 48–60, 60–72, 72–84, 84–96 h post dose. At each time interval, the amount of lymph collected was determined gravimetrically. Lymph samples were stored at 4°C for up to 12 h and at −80°C for long-term storage.

Aliquots of lymph and serum were shipped on dry ice to the bioanalytical site where they were stored at approximately −80°C until analyzed.

### ELISA Assay for BIL

BIL concentrations in serum and lymph were determined by ELISA performed by the bioanalytical testing facility Charles River Laboratories Preclinical Services (Senneville, Quebec, Canada) as described previously for rat serum (27). The assay quantitation range for rat serum was 20 pM to 500 pM. Assay quantitation range for sheep serum was from 20 pM to 500 pM. A 10% dilution of serum was used as a surrogate matrix for the sheep lymph. The quantitation range for sheep lymph was also 20 to 500 pM. Samples above the upper limit of quantitation were diluted and reanalyzed to gain results within the calibrated range. The assays were validated (serum) or verified (lymph) using quality control samples at three different concentrations. The measure of precision (relative standard deviation) and the measure of accuracy (relative error) for the serum-assays were ≤15% across the quantitation range. For the lymph-assay, the relative standard deviation and relative error were ≤20% across the quantitation range. BIL was shown to be stable in frozen serum and lymph.

### RIA Assay for Insulin Lispro

Insulin lispro in rat serum was determined using an RIA for insulin lispro at EMD Millipore (St Charles, MO). The assay quantitation range was 10 pM to 5000 pM. Samples with concentrations higher than 5000 pM were diluted before analyses to yield a concentration within the quantitation range.

### Pharmacokinetic Data Analyses

Noncompartmental pharmacokinetic parameters for the rat study were determined using WinNonlin® professional v5.3 (Pharsight, St. Louis, MO, USA) and for the sheep study using Watson^TM^ (version 7.4.2 PRD872, Thermo Fisher Scientific, Inc). Concentrations below the lower limit of quantitation were assigned a value of zero and the zero result used in the calculation of means and standard deviation using MicroSoft® Office® Excel® 2010 (version 14.0.; Redmond, WA). Descriptive statistics were calculated as appropriate. SigmaPlot® v11 (Systsat Software, IC., San Jose, CA, USA) and Excel® were used for the graphics.

## RESULTS

### Hydrodynamic Size

Using DLS to measure hydrodynamic size, BIL displayed a hydrodynamic diameter of 7.6 nm (±0.6 nm), whereas insulin lispro displayed a hydrodynamic diameter considerably smaller, 2.1 nm (±0.3 nm). Although the MW of BIL is only 26 kDa, the hydrodynamic diameter is similar to human serum albumin, which has a MW of 67 kDa (~7.2 nm) (28). The hydrodynamic size for insulin (MW = 6 kDa) was 2.1 nm, and the hydrodynamic size for leptin (MW = 16 kDa) was 4.3 nm.

### Pharmacokinetics in 5/6-Nephrectomized Rats

**Fig. 1:**
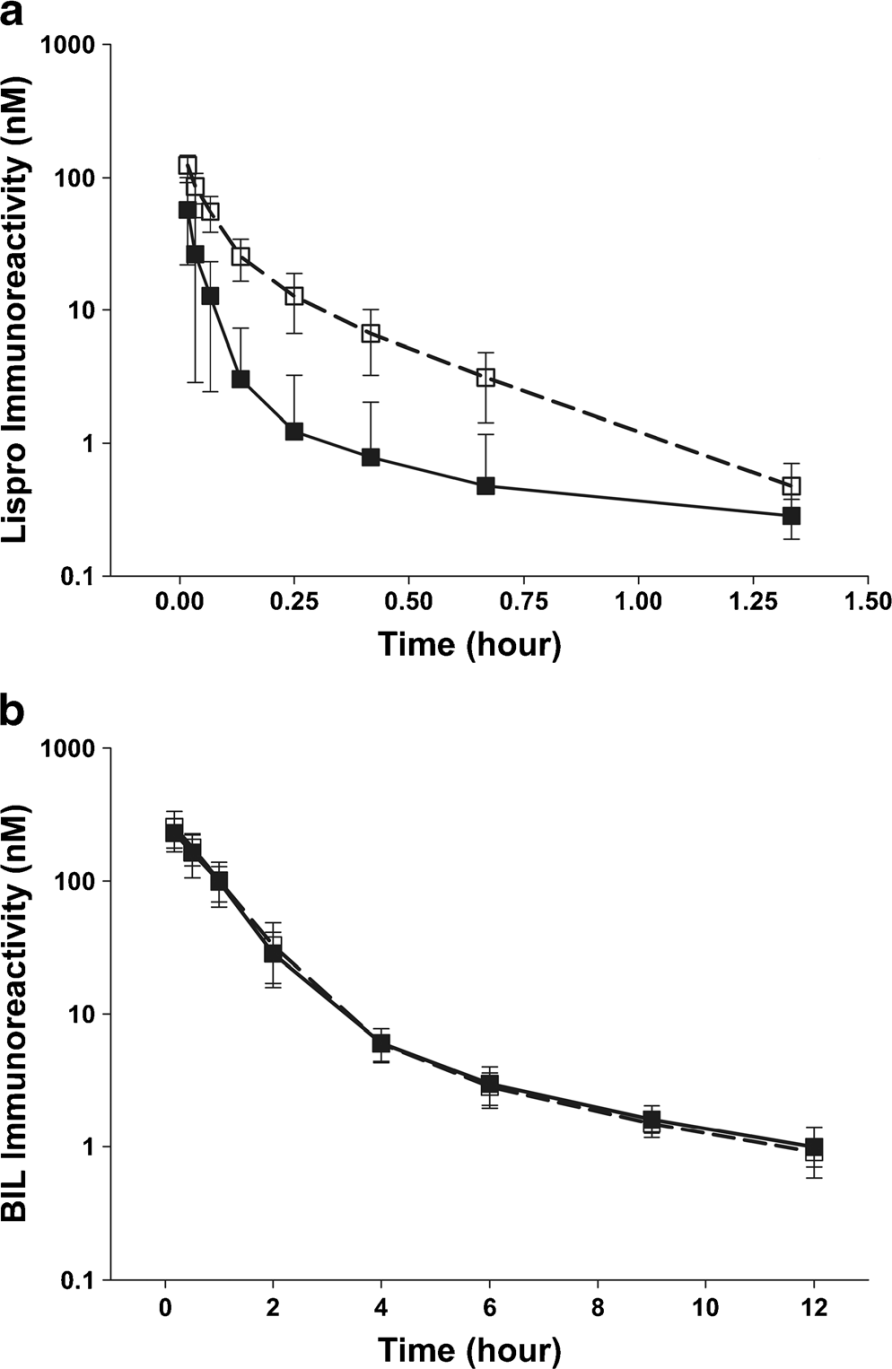
Pharmacokinetic profiles for BIL (**a**) and insulin lispro (**b**) in sham and 5/6-nephrectomized rats. BIL was tested in 2 independent studies and insulin lispro was tested in 3 independent studies. BIL (**a**): sham groups (*n* = 13, closed squares); 5/6-nephrectomized groups (*n* = 16, open squares). Insulin lispro (**b**): sham groups (*n* = 24, closed squares); 5/6-nephrectomized groups (*n* = 28, open squares). All data are represented as mean ± SD. Abbreviations: *BIL* basal insulin peglispro.

After IV administration to sham rats, the C_0_ and AUC of BIL were higher than the C_0_ and AUC for insulin lispro (Fig. 1; Table II). The clearance of BIL was slower than that for insulin lispro in the sham rats and the volume of distribution was lower. In sham rats, the clearance of BIL was reduced ~65-fold versus insulin lispro, from 2.24 L/hr/kg to 0.035 L/hr/kg. In 5/6-nephrectomized rats, where the contribution of renal clearance should be minimal, the clearance of BIL was ~20-fold less than the clearance of insulin lispro, but was similar to sham rats administered with BIL. For insulin lispro, the clearance in the 5/6-nephrectomized rats was reduced 3.3-fold when compared to sham rats suggesting an impact on clearance by renal impairment, while the clearance of BIL was similar in sham and 5/6-nephrectomized rats.

**Table II:** Pharmacokinetic Parameters for IV-injected 10 nmol/kg Insulin Lispro and BIL in 5/6-nephrectomized Sprague–Dawley Rats

Mean (SEM)	Insulin Lispro		BIL	
Model	5/6	Sham	5/6	Sham
C_0_ (nM)	184 (38)	123 (31)	295 (22)	239 (22)
AUC_0-∞_ (nM × hour)	15 (1.4)	4 (0.5)	305 (16)	287 (19)
CL (L/hour/kg)	0.67 (0.064)	2.24 (0.262)	0.033 (0.0017)	0.035 (0.0023)
Vd_ss_ (L/kg)	0.13 (0.022)	0.42 (0.087)	0.050 (0.004)	0.059 (0.007)

### Pharmacokinetics in Non-LDC Sheep

The pharmacokinetics of BIL in non-LDC sheep were determined after IV administration of 20 nmol, 40 nmol, and 120 nmol of BIL as well as after SC administration of 120 nmol. After IV administration, exposure increased linearly as the dose increased from 20 nmol to 120 nmol (Table III). The mean half-life of BIL ranged from ~2–3 h after IV administration in non-LDC sheep and was more than doubled to ~8 h after SC administration in non-LCD sheep (Table IV).

**Table III:** Pharmacokinetic Parameters in Serum After IV Administration of BIL to non-LDC Sheep

Parameter	BIL (Mean ± SD)		
Number (N)	3	4	3
Dose (nmol)	20	40	120
AUC_0-t_ (pM × hours)	12566.7 ± 1800.9	40075.0 ± 12458.3	111666.7 ± 15044.4
T_1/2_ (hours)	1.6 ± 0.1	2.8 ± 0.3	3.3 ± 1.2
CL (mL/min/kg)	0.70 ± 0.09	0.53 ± 0.21	0.47 ± 0.06
Vd_ss_ (L/kg)	0.05 ± 0.01	0.04 ± 0.01	0.07 ± 0.04
C_0_ (pM)	11133 ± 1234	27900 ± 6124	60400 ± 1039

**Table IV:** Pharmacokinetic Parameters in Serum After Administration of 120-nmol BIL to Sheep

Parameter (Units)	BIL (Mean ± SD)			
Number (N)	4	5	4	3
Route	SC (LDC)	SC (non-LDC)	IV (LDC)	IV (non-LDC)
AUC_0-t_ (pM × hours)	2594.5 ± 2563.6	142960.0 ± 83820.7	139500.0 ± 60693.2	111666.7 ± 15044.4
T_1/2_ (hours)	4.6 ± 4.0^a^	7.5 ± 5.1	5.7 ± 2.1	3.3 ± 1.2
CL (mL/min/kg)	NA	NA	0.37 ± 0.12	0.47 ± 0.06
Vd_ss_ (L/kg)	NA	NA	0.04 ± 0.01	0.07 ± 0.04
C_0_ (pM)	NA	NA	70175.0 ± 13600.1	60400 ± 1039.2
T_max_ (hours)	2.3 ± 1.0	2.4 ± 0.9	NA	NA
C_max_ (pM)	415.3 ± 384.0	18766.0 ± 7833.3	NA	NA
% bioavailability	1.7	123	NA	NA
AUC_LDC_/AUC_intact_	0.018		1.25	

^a^ *N* = 3

### Pharmacokinetics of BIL in LDC and Non-LDC Sheep

**Fig. 2:**
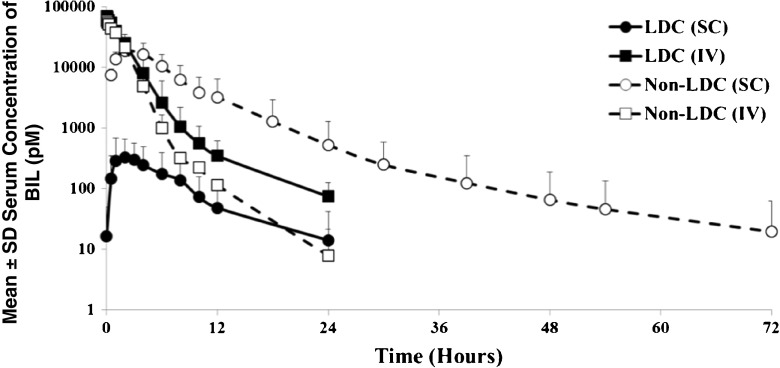
Mean (SD) serum concentrations vs time for LDC and non-LDC sheep after administration of 120 nmol BIL either SC or IV. The mean is for 3 to 5 sheep per group. Abbreviations: *BIL* basal insulin peglispro, *LDC* lymph duct cannulated, *IV* intraveneous, *SC* subcutaneous, *SD* standard deviation.

The mean serum concentrations of BIL after a single SC dose of 120 nmol BIL showed 50 to 100-fold higher concentrations in non-LDC sheep compared to LDC sheep (Fig. 2). The AUC for 120 nmol SC administration in LDC sheep was <2% of the AUC in non-LDC sheep, but after 120 nmol IV administration the AUCs were similar in LDC and non-LDC sheep. The absolute bioavailability after SC administration to non-LDC sheep was 123.0%, but decreased to 1.7% for the LDC sheep administered the same dose (Table IV).

**Fig. 3:**
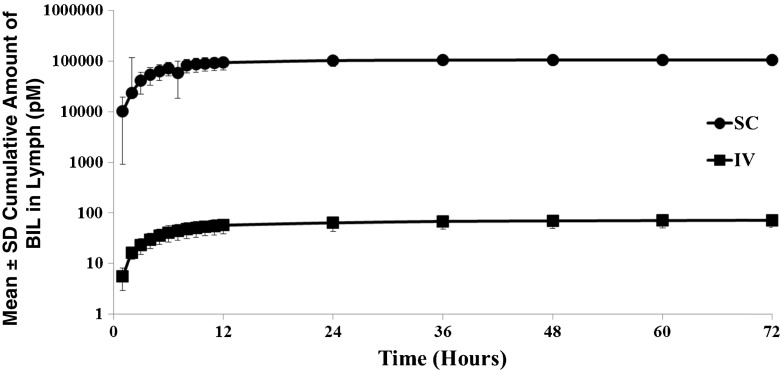
Mean (SD) percent of dose recovered in lymph from LDC and non-LDC sheep after administration of 120 nmol of BIL either SC or IV. The mean is for 3 to 5 sheep per group. Abbreviations: *BIL* basal insulin lispro, *IV* intravenous, *LDC* lymph duct cannulated, *SC* subcutaneous, *SD* standard deviation.

In LDC sheep, 88% (±19% standard deviation [SD]; 61%–102% range) of BIL was recovered in the peripheral lymph after SC dosing compared to 0.06% (±0.02%; 0.04–0.08%) recovered after IV dosing. Figure 3 shows the cumulative amount of BIL collected in lymph after a single 120 nmol IV or SC dose.

## DISCUSSION

The addition of a single linear 20-kDa PEG to insulin lispro resulted in a molecule of insulin lispro with a hydrodynamic size of 7.6 nm rendering the molecule a size similar to or greater than albumin. The results presented herein demonstrate that the addition of a 20-kDa PEG to insulin lispro leads to changes in both the absorption and clearance of insulin lispro. The renal clearance of BIL compared to insulin lispro decreased in the rat. Renal impairment did not appear to alter the clearance of BIL in the 5/6-nephrectomized rat model. The differences in clearance seen in the 5/6-nephrectomized rats between BIL and insulin lispro can be attributed to a reduction in renal clearance of BIL. In total, the rat data suggests that PEGylation of insulin lispro increases the hydrodynamic size of insulin lispro beyond that associated with molecular weight changes, contributing to a decreased clearance, which contributes to the prolonged duration of action of BIL.

In a previously reported study in diabetic rats, the clearance of total insulin was decreased 13-fold after BIL administration compared to after insulin lispro administration and the AUC was increased 10-fold after BIL administration (29). The T_max_ for total insulin after administration of BIL was approximately 24-fold longer than that after insulin lispro administration. These data indicated that in rats, both absorption and clearance were impacted by the PEGylation of insulin lispro to yield BIL.

The absorption of BIL was predominately lymphatic absorption, which may have affected the rate of absorption. The delay in absorption could be due to the requirement for lymphatically transported drugs to pass through the lymphatics prior to reaching the systemic circulation and/or related to a slower transport through the interstitium to reach the lymphatic capillaries. In general, however, lymphatic transport is rapid, and previous studies exploring intestinal lymphatic transport of, for example, lipophilic small molecules in dogs (where absorption is rapid) have shown complete transport through the lymphatic in approximately 1 to 2 h (30). It seems likely, therefore, that the effect on absorption rate here reflects slower transport across the interstitial space to the lymphatic capillaries rather than slowed transport through the lymphatics, although the data are not definitive. Similar T_max_ data for BIL absorption into the blood in non-LDC and LDC sheep suggests that absorption rates into blood and lymph are similar, providing support for the suggestion that transport through the lymph is rapid. Several animal models used in measuring the contribution of the lymphatic system to parenterally-administered macromolecules have demonstrated that higher MW molecules are more readily absorbed through the lymphatic system following SC injection (14,20,31–33), but no data are available on hydrodynamic size and lymphatic absorption. The LDC-sheep model showed that the large hydrodynamic size of BIL increases the role of the lymphatic system in absorption of BIL, beyond that associated with molecular weight changes, after SC administration.

Because one assumption of the bioavailability calculation in the sheep model is PK linearity, non-LDC sheep were administered increasing doses of BIL. The increase in mean serum concentrations and AUC for each dose indicated that the PK of BIL in sheep was linear and the LDC model is valid. The increase in half-life observed with SC (~8 h) versus IV (~3 h) injection in non-LDC sheep indicated absorption rate-limited (ie, flip-flop) pharmacokinetics after SC administration.

After SC administration, when the lymph was collected, the AUC (exposure/bioavailability) and mean serum concentrations of BIL in LDC sheep were greatly reduced compared with non-LDC sheep reflecting the fact that the dose was collected in the lymph and removed before reaching the systemic circulation. However, after IV administration, when lymph was collected, the AUCs were similar n LDC sheep and non-LDC sheep, with <1% of the dose recovered in the lymph of LDC sheep. Access to the peripheral lymphatics via equilibration across peripheral capillary beds (rather than direct access at the injection site) was therefore low, suggesting that BIL is primarily absorbed via the lymphatic system after SC administration.

The increase in hydrodynamic diameter of BIL due to the 20-kDa PEG moiety (7.6 nm vs 2.1 nm for insulin lispro) was similar to the increase observed by addition of 20-kDa PEG to 14.3-kDa egg white lysozyme (9.6 nm vs. 4.0 nm) (28,34,35). This marked increase in hydrodynamic size, relative to the MW of BIL, suggests that the PEG is a conformationally dynamic random-coil structure; thus, decoupling size from MW (28,36). This dynamic movement of the PEG and the resulting increase in hydrodynamic size helps to explain the minimal renal clearance of 20-kDa mono-PEGylated insulin lispro species based on sieving coefficient analysis performed with molecules having different Stokes-Einstein radii (37). Similar to the results in the 5/6-nephrectomized rat model, the PK properties of BIL have been shown to be unaffected by renal insufficiency in humans (38), whereas insulin undergoes substantial renal clearance (39–41). Previous studies in rats showed that after SC administration only 2.5% of a dose of BIL is excreted unchanged in urine and 11% after IV administration (27).

**Fig. 4:**
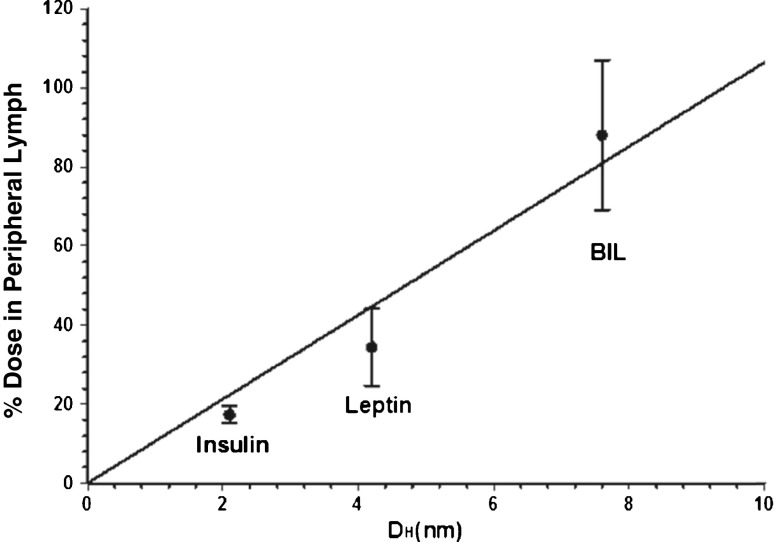
The percent recovery of BIL in peripheral lymph is greater than that of both insulin and leptin which have smaller hydrodynamic sizes. The line represents the linear correlation. *BIL* basal insulin peglispro, D_H_ = 7.6 nm (±0.6 nm), Insulin D_H_ = 2.1 nm, Leptin D_H_ = 4.2 nm.

The hydrodynamic size of BIL likely accounts for much of the increase in lymphatic absorption of BIL in the current study (88%) versus the amount of recovered insulin observed in a previous study (17%) (13). The correlation observed between hydrodynamic size of BIL with percent dose recovered in peripheral lymph shows that increasing hydrodynamic size of insulin lispro with the addition of 20-kDa PEG can readily shift absorption of insulin to lymphatic capillaries versus blood capillaries by altering paracellular transport in the microvascular beds (14,17,18,20,31–33). Previous studies have correlated the percent of lymphatic absorption with the MW of administered proteins (14,20,31–33). However, the results from this study suggest that for exogenously administered insulin a better indicator of lymphatic absorption may be hydrodynamic size. The percent of lymphatic absorption in the LDC-sheep model of insulin, leptin and BIL are plotted versus the hydrodynamic size in Fig. 4.

## CONCLUSION

These results suggest that the increased hydrodynamic size of insulin lispro with the addition of 20-kDa PEG in BIL likely alters paracellular transport leading to a slowing of the rate of absorption of BIL into the serum/plasma compartment and that this occurs simultaneous with a shift in the absorption process from absorption into the blood capillaries to absorption via the lymph capillaries. It seems likely, however, that the slower rate of absorption of BIL when compared with insulin lispro reflects slower diffusion or convection across the interstitial injection site to either blood or lymph capillaries, rather than intrinsically slow lymphatic transport. At the same time, the addition of 20-kDa PEG significantly reduces renal clearance compared with that of insulin lispro, resulting in enhanced blood levels. PEGylation, therefore, provides a useful means to increase the systemic exposure of insulin after SC administration by changes in both absorption and clearance.

## ABBREVIATIONS

AUC_0-t_: Area under the serum concentration-time curve from 0 to time of the last sample with a quantifiable concentration
BIL: Basal insulin peglispro
C_o_: Serum concentration extrapolated to time 0 after IV administration
CL: Total body clearance from serum
C_max_: Maximum observed serum concentration
D: Diffusion constant
DLS: Dynamic light scattering
D_H_: Apparent hydrodynamic diameter
ELISA: Enzyme-linked immunosorbant assay
LDC: Lymph duct cannulated
MW: Molecular weight
OD_276_: Optical density at 276 nm
Pa: Pascal
PBS: Phosphate buffered saline
PEG: Polyethylene glycol
PK: Pharmacokinetic
IV: Intravenous
k_B_: Boltzmann constant
η: Viscosity
R_h_: Apparent hydrodynamic radius
RIA: Radio-immunoassay
rpm: Revolution per minute
SC: Subcutaneous
SD: Standard deviation
T_1/2_: Terminal elimination half-life
T: Absolute temperature
T_max_: Time at the maximum concentration in serum
V_dss_: Volume of distribution at steady state
